# Reactive oxygen species explicit dosimetry to predict tumor growth for benzoporphyrin derivative-mediated vascular photodynamic therapy

**DOI:** 10.1117/1.JBO.25.6.063805

**Published:** 2020-01-07

**Authors:** Tianqi Sheng, Yihong Ong, Wensheng Guo, Timothy C. Zhu

**Affiliations:** a University of Pennsylvania, Department of Radiation Oncology, Philadelphia, Pennsylvania, United States; b University of Pennsylvania, Department of Biostatistics and Epidemiology, Philadelphia, Pennsylvania, United States

**Keywords:** photodynamic therapy (PDT), explicit dosimetry, singlet oxygen, PDT dose, benzoporphyrin derivative-mediated PDT, reactive oxygen species, *in vivo* mouse study

## Abstract

Photodynamic therapy (PDT) is a well-established treatment modality for cancer and other malignant diseases; however, quantities such as light fluence and PDT dose do not fully account for all of the dynamic interactions between the key components involved. In particular, fluence rate (ϕ) effects, which impact the photochemical oxygen consumption rate, are not accounted for. In this preclinical study, reacted reactive oxygen species (${\left[\mathrm{ROS}\right]}_{\mathrm{rx}}$) was investigated as a dosimetric quantity for PDT outcome. The ability of ${\left[\mathrm{ROS}\right]}_{\mathrm{rx}}$ to predict the cure index (CI) of tumor growth, $\mathrm{CI}=1-k/k_{\mathrm{ctr}}$, where k and $k_{\mathrm{ctr}}$ are the growth rate of tumor under PDT study and the control tumor without PDT, respectively, for benzoporphyrin derivative (BPD)-mediated PDT, was examined. Mice bearing radiation-induced fibrosarcoma (RIF) tumors were treated with different in-air fluences (Φ=22.5 to $166.7\;J/{\mathrm{cm}}^{2}$) and in-air fluence rates ($\phi_{\text{air}}=75$ to $250\;\mathrm{mW}/{\mathrm{cm}}^{2}$) with a BPD dose of 1  mg/kg and a drug-light interval (DLI) of 15 min. Treatment was delivered with a collimated laser beam of 1-cm-diameter at 690 nm. Explicit measurements of in-air light fluence rate, tissue oxygen concentration, and BPD concentration were used to calculate for ${\left[\mathrm{ROS}\right]}_{\mathrm{rx}}$. Light fluence rate at 3-mm depth ($\phi_{3\;\mathrm{mm}}$), determined based on Monte-Carlo simulations, was used in the calculation of ${\left[\mathrm{ROS}\right]}_{\mathrm{rx}}$ at the base of tumor. CI was used as an endpoint for three dose metrics: light fluence, PDT dose, and ${\left[\mathrm{ROS}\right]}_{\mathrm{rx}}$. PDT dose was defined as the product of the time-integral of photosensitizer concentration and $\phi_{3\;\mathrm{mm}}$. Preliminary studies show that ${\left[\mathrm{ROS}\right]}_{\mathrm{rx}}$ best correlates with CI and is an effective dosimetric quantity that can predict treatment outcome. The threshold dose for ${\left[\mathrm{ROS}\right]}_{\mathrm{rx}}$ for vascular BPD-mediated PDT using DLI of 15 min is determined to be 0.26 mM and is about 3.8 times smaller than the corresponding value for conventional BPD-mediated PDT using DLI of 3 h.

## 1 Introduction

Photodynamic therapy (PDT) is a treatment modality for cancer and other localized diseases. PDT is not only “dynamic” but also multifaceted.^1^^,^^2^ PDT incorporates light, photosensitizer, and oxygen to create reactive oxygen species (ROS) to kill cells. Unlike radiotherapy and chemotherapy, PDT causes fewer side effects, as it does not involve ionizing radiation and can be well-localized.^3^^,^^4^ PDT is uniquely advantageous compared to other treatment modalities, as it is also associated with fast postoperative recovery and better cosmetic outcome. However, widespread use of PDT has been stymied due to the difficulty in accurately quantifying the dose. Furthermore, assessment of PDT efficacy is difficult due to the lack of a well-defined dose metric that accurately predicts biological response. ROS are acceptable to be the cytotoxic agents causing therapeutic outcome in PDT. Direct detection of ROS can provide the most accurate quantity to guide treatments and predict treatment outcomes. However, *in vivo* detection of ROS during clinical PDT is very challenging due to its weak signal and short lifetime. To overcome this, a macroscopic reactive oxygen species explicit dosimetry (ROSED) model was recently developed to calculate for the accumulated reacted ROS concentration (${\left[\mathrm{ROS}\right]}_{\mathrm{rx}}$) that is predictive of PDT treatment outcome.^5^^–^^8^

Benzoporphyrin derivative monoacid ring A (BPD-MA, trademark Visudyne^®^) is a commonly used photosensitizer that has been approved by the U.S. Food and Drug Administration for the treatment of wet age-related macular degeneration.^9^ Using a macroscopic ROSED model of light fluence (rate), BPD drug concentration, and tissue oxygen concentration ([O23]), ${\left[\mathrm{ROS}\right]}_{\mathrm{rx}}$ can be determined to evaluate its effectiveness as a dosimetrical predictor for BPD-mediated PDT outcome. Vascular-targeted PDT can be achieved using a short (15 min) drug-light interval (DLI), defined as the time interval between the PS drug injection and the start of PDT treatment.^10^^,^^11^ By inducing vascular shutdown, nutrient supply and removal of metabolic waste is halted, which results in radiation-induced fibrosarcoma (RIF) tumor cell death. This is beneficial as targeting tumor vasculature is easier to access, more efficient in cancer cell killing, and has a lower likelihood of developing drug resistance.

This study, to our knowledge, is the first study to investigate the relationship between various dose metrics (fluence, PDT dose, and ${\left[\mathrm{ROS}\right]}_{\mathrm{rx}}$) and cure index (CI) at 14 days in an *in vivo* mouse model for BPD-mediated vascular PDT. ROSED was performed to evaluate the treatment outcomes of BPD-mediated vascular PDT in mice bearing RIF tumors. The major photochemical parameters in the macroscopic ROSED model have been investigated and determined for the photosensitizer BPD-MA for DLI of 3 h^12^^,^^13^ and are found to be similar for DLI of 15 min.^14^

## 2 Materials and Methods

### 2.1 Tumor Model

**Fig. 1:**
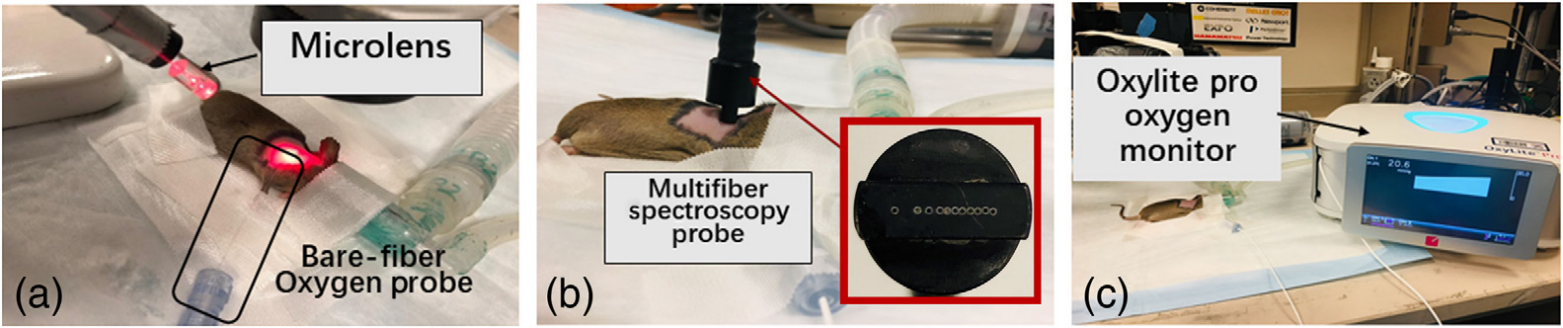
Experiment setup with the (a) multifiber contact spectroscopy probe, tissue ${\mathrm{pO}}_{2}$ was recorded before and during PDT treatment. (b) A handheld broadband reflectance spectroscopy contact probe was used to measure the optical properties and drug concentration before and after PDT. (c) Oxylite prooxygen monitor with a fluorescence-based bare-fiber oxygen probe (Oxford Optronix, Oxford, UK)

RIF cells were cultured and 30  μl were injected at $1\times 10^{7}\text{ cells}/\mathrm{ml}$ in the right shoulders of 6- to 8-week-old female C3H mice (NCI-Frederick, Frederic), as described previously.^5^^,^^15^^–^^17^ The resulting RIF tumors are subcutaneous. Animals were under the care of the University of Pennsylvania Laboratory Animal Resources. All studies were approved by the University of Pennsylvania Institutional Animal Care and Use Committee. Tumors were treated when they were ∼3 to 5 mm in diameter. The fur of the tumor region was clipped prior to cell inoculation, and the treatment area was depilated with Nair (Church and Dwight Co., Inc., Ewing, New Jersey) at least 24 h before measurements. Mice were provided a chlorophyll-free (alfalfa-free) rodent diet (Harlan Laboratories Inc., Indianapolis, Indiana, United States) starting for at least 10 days prior to treatment to eliminate the fluorescence signal from chlorophyll-breakdown products, which have a similar emission range to the BPD fluorescence spectra used to determine the concentration of BPD in the tumor. During the whole treatment, mice were kept under anesthesia on a heat pad at 38°C [see Fig. 1(a)].

### 2.2 PDT Treatment Conditions

Treatment delivery was done using an optical fiber with a microlens attachment coupled to a diode laser. A 690-nm laser (B&W Tek Inc., Newark, Delaware) was used for PDT after a 15-min DLI. The in-air fluence rate ($\phi_{\text{air}}$) is defined as the calculated irradiance determined by the laser power divided by the treatment area (1-cm diameter spot size). The in-air fluence was calculated by multiplying the in-air fluence rate by the treatment time. RIF tumor-bearing mice with no photosensitizer and no light excitation were used as controls (n=5). The photochemical parameters are summarized in Table 1.

**Table 1:** Photochemical parameters for BPD based on Refs. 18 and 19.

Photochemical parameter	Definition	Value	References
ε (${\mathrm{cm}}^{-1}\;\mu M^{-1}$)	Photosensitizer extinction coefficient	0.0783	18 and 19
δ (μM)	Low-concentration correction	33	18 and 19
β (μM)	Oxygen quenching threshold concentration	11.9	18 and 19
σ ($\mu M^{-1}$)	Specific photobleaching ratio	$(1.8\pm 0.3)\times 10^{-5}$	18 and 19
ξ (${\mathrm{cm}}^{2}\;{\mathrm{mW}}^{-1}\;s^{-1}$)	Specific oxygen consumption rate	$(55\pm 15)\times 10^{-3}$	18 and 19
g ($\mu M\;s^{-1}$)	Macroscopic maximum oxygen supply rate	1.7±0.4	18 and 19
${\left[\mathrm{ROS}\right]}_{\mathrm{rx},\mathrm{sh}}$ (mM)	Singlet oxygen threshold dose for tumor regrowth	0.26±0.05	This study
${\left[D\right]}_{0}$ ($\mu M\;J/{\mathrm{cm}}^{2}$)	PDT dose, the product of the drug concentration, and light fluence	7.5±1.0	This study

### 2.3 Photodynamic Therapy Treatment Protocol

BPD (trademark Visudyne^®^) at a dosage of 1  mg/kg was injected through the mouse tail vein as described previously.^16^^,^^19^ At a 15-min DLI, superficial irradiation of the tumor was performed with a 690-nm diode laser with a maximum output power of 8 W (B&W Tek Inc., Newark, Delaware). A microlens fiber was coupled to the laser to irradiate the tumor uniformly [see Fig. 1(a)]. Mice were treated with in-air fluence rates ($\phi_{\text{air}}$) of 75 to $250\;\mathrm{mW}/{\mathrm{cm}}^{2}$ and total in-air fluences of 22.5 to $166.7\;J/{\mathrm{cm}}^{2}$ to induce different PDT outcomes and to assess the reciprocity between BPD concentration and light dose. The “in-air fluence rate” is defined as the calculated irradiance determined by laser power divided by the treatment area. The “in-air fluence” was calculated by multiplying the “in-air fluence rate” by the treatment time. Animals were assigned to six light dose groups (see Table 2). RIF tumor-bearing mice that received neither light irradiation nor BPD were used as controls.

**Table 2:** In-air light fluence, in-air light fluence rate $\phi_{\text{air}}$, photosensitizer concentrations pre-PDT ${\left[\mathrm{BPD}\right]}_{\mathrm{pre}}$, PDT dose at 3-mm depth, reacted oxygen species concentration ${\left[\mathrm{ROS}\right]}_{\mathrm{rx}}$ at 3-mm depth, tumor regrowth rate k, and CI for each PDT treatment group. Number of mice per group is shown in the second column.

Group #	# mice	In-air fluence ($J/{\mathrm{cm}}^{2}$)	$\phi_{\text{air}}$ ($\mathrm{mW}/{\mathrm{cm}}^{2}$)	Time (s)^a^	${\left[\mathrm{BPD}\right]}_{\mathrm{pre}}$ (μM)	[O23] (μM)	Fluence^b^ ($J/{\mathrm{cm}}^{2}$)	PDT dose^c^ ($\mu M\;J/{\mathrm{cm}}^{2}$)	${\left[\mathrm{ROS}\right]}_{\mathrm{rx}}$^d^ (mM)	k (1/days)	CI^e^ variance count	$\mathrm{CI}=1-k/k_{\mathrm{ctr}}$
1	2	22.5	75	300	0.24±0.01	17.6±5.9	21.4±0.8	5.0±0.3	0.11±0.04	0.41±0.03	0/2	0.04±0.03
2	3		75	300	0.19±0.05	6.6±2.3	22.3±0.2	4.2±1.0	0.04±0.01	0.41±0.03	0/3	0.02±0.27
3	3	30.0	75	400	0.18±0.02	29±18	28.1±2.9	5.0±0.7	0.16±0.04	0.390±0.003	0/3	0.07±0.18
4	3		75	400	0.27±0.03	31±14	30.2±4.3	7.9±0.8	0.29±0.04	0.15±0.06	1/3	0.63±0.04
5	2	45	75	600	0.28±0.06	39.1±0.7	43.6±9.0	10.7±0.6	0.46±0.03	0	2/2	1±0.00
6	4		75	600	0.20±0.05	19.2±1.6	44.1±7.6	8.7±1.7	0.24±0.03	0.26±0.07	0/4	0.38±0.04
7	2	50	75	666	0.15±0.02	19.1±0.7	44.5±4.5	7.7±1.2	0.20±0.02	0.29±0.06	0/2	0.30±0.03
8	5		75	666	0.20±0.01	22±12	45.9±5.4	9.5±0.9	0.28±0.06	0.19±0.05	1/5	0.55±0.06
9	5	93.4	200	467	0.13±0.03	32±11	94.6±6.1	11.3±1.6	0.44±0.09	0	5/5	1±0.00
10	5	166.7	250	667	0.15±0.03	17.0±4.0	168.3±5.4	29.9±5.3	0.68±0.09	0	5/5	1±0.00
11	5	Control	0	0	0	0	0	0	0	0.43±0.05	—	0

a Total treatment time.

b Light fluence is determined at 3-mm depth based on measured mean in-air light fluence rate at surface and $\phi /\phi_{\text{air}}$, Eq. (6), using the mean tissue optical properties: $\mu_{a}=6.9\;{\mathrm{cm}}^{-1}$, $\mu_{s}^{\prime }=11\;{\mathrm{cm}}^{-1}$.^18^ The actual measured in-air fluence rate at surface can be ±10% different from the nominal values and they are accounted in the fluence ($J/{\mathrm{cm}}^{2}$) at 3 mm.

c PDT dose is defined as the time integral of the product of the photosensitizer concentration and the light fluence rate (ϕ), $\left[S_{0}](t\right)$ is calculated using the parameters in Table 1.

d ${\left[\mathrm{ROS}\right]}_{\mathrm{rx}}$ is calculated using Eq. (5) using the parameters listed in Table 1.

e CI variance count gives number of mice with no tumor regrowth after PDT, out of the total number of mice in the group.

### 2.4 Oxygen Measurements

The *in vivo* tissue oxygen partial pressure ${\mathrm{pO}}_{2}$ was measured during PDT treatment using a phosphorescence-based O23 probe (OxyLite Pro, Oxford Optronix, Oxford, United Kingdom). A bare-fiber-type probe (NX-BF/O/E, Oxford Optronix, Oxford, United Kingdom) was placed inside the tumor at a 3-mm depth from the treatment surface. The O23 concentration ([O23]) was calculated by multiplying the measured ${\mathrm{pO}}_{2}$ with the O23 solubility in tissue, which is 1.295  μM/mmHg.^20^^,^^21^ Measured [O23]0 and [O23](t) was used to calculate for ${\left[\mathrm{ROS}\right]}_{\mathrm{rx}}$ using the macroscopic ROSED model.^20^^,^^22^

### 2.5 Measurement of BPD Concentration

Following the DLI of 15 min, measurements of light fluence rate, photosensitizer concentration, and [O23] were performed. BPD fluorescence spectra were obtained using a custom-made multifiber contact probe before and after PDT.^1^ The probe was connected to a 405-nm laser (Power Technology Inc., Little Rock, Arkansas) for the fluorescence excitation of BPD and a multichannel CCD spectrograph (InSpectrum, Princeton Instruments, Trenton, New Jersey) for the collection of the fluorescence spectra. The *in vivo* photosensitizer concentration was obtained by comparing the measured BPD spectra with those of phantoms with known photosensitizer concentrations. The attenuation of the fluorescence signal due to the light absorption and scattering by tissues was corrected by applying an empirical correction factor described elsewhere.^19^ The accuracy of *in vivo* measurements was validated by *ex vivo* measurements in separate mice.

### 2.6 *Ex Vivo* Validation of BPD Concentration

**Fig. 2:**
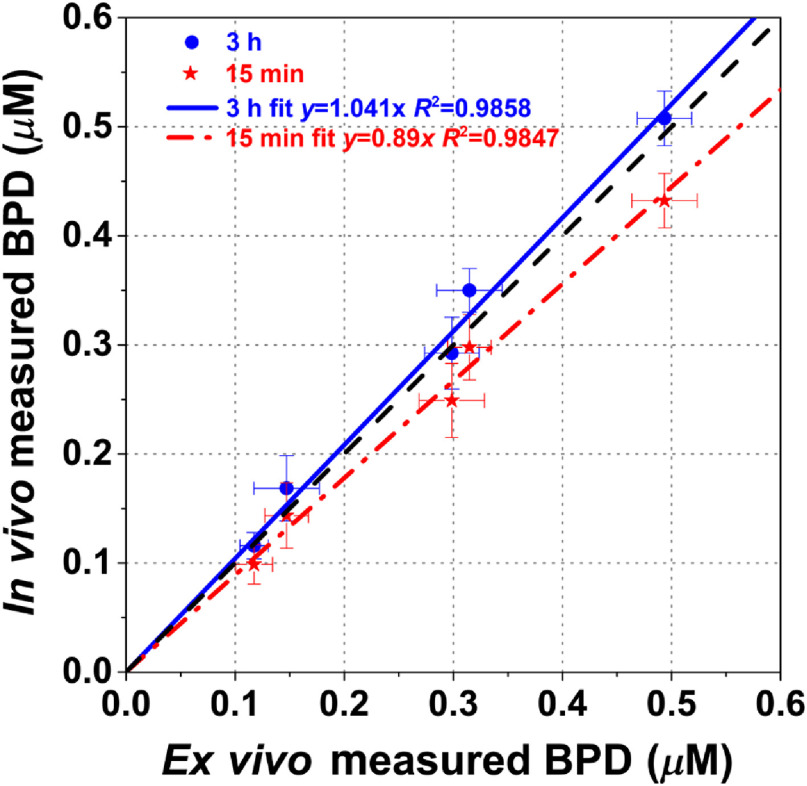
Comparison between *in vivo* and *ex vivo* measured BPD concentrations. Solid line is a linear fit for *ex vivo* versus *in-vivo* measured BPD concentration at 3 h. Dash-dot line is a linear fit to 15 min results. *Ex vivo* measurements were made at 3 h only since BPD concentration at 15 min is not detectable *ex vivo*. Dashed line is for y=x.

*In vivo* fluorescence measurements of the photosensitizer concentration as described above were performed for all tumors before PDT. To evaluate the accuracy of the *in vivo* fluorescence measurements, *ex vivo* measurements of the BPD concentration were performed in separate set of mice and compared with the BPD concentration determined from *in vivo* measurements. All five mice were administered BPD at different concentrations between (0.25 to 1.25  mg/kg). *In vivo* fluorescence measurements were taken from each mouse at 15 min and 3 h after BPD administration. After fluorescence measurements at 3-h time point were taken, mice were euthanized and the tumors were excised, protected from light, and stored at −80°C. For *ex vivo* analyses, homogenized solutions of the tumors were prepared using Solvable (PerkinElmer, Waltham, Massachusetts). The fluorescence of the homogenized sample was measured by a spectrofluorometer (FluoroMax-3; Jobin Yvon, Inc.) with an excitation wavelength of 405 nm and an emission range from 630 to 750 nm with an emission maximum at 667 nm. The photosensitizer concentration in the tissue was calculated based on the change in fluorescence resulting from the addition of a known amount of BPD to each sample after its initial reading. The *in vivo* measurements were correlated to *ex vivo* data using a linear fit to examine their agreement based on the goodness of the fit ($R^{2}$) (see Fig. 2). The *ex vivo* measurements of BPD concentration were compared to those obtained *in vivo* using the contact probe method to evaluate for the accuracy of the *in vivo* acquired BPD concentrations. The linear fit to 3 h DLI results (shown as a solid line) shows close agreement between the *in vivo* and *ex vivo* BPD concentrations, y=1.041x, with a fitting goodness of $R^{2}=0.9858$; similarly, linear fit to 15 min DLI results (shown as a dash-dot line) shows reasonable agreement between the *in vivo* and *ex vivo* BPD concentrations, y=0.89x, with a fitting goodness of $R^{2}=0.9847$. The dashed line represents the line for y=x, if the two measurements were completely in agreement.

### 2.7 Tumor Regrowth Rate Analysis

Tumor volumes were tracked daily, for 14 days, after PDT. Width (a) and length (b) were measured with slide calibers, and tumor volumes (V) were calculated using $V=\pi \times a^{2}\times b/6$.^23^ Tumor regrowth factor (k) was calculated by the best exponential fit [with a form $f(d)=Ae^{kd}$] to the measured volumes over the days (d). CI was calculated for each treatment group as

$$\mathrm{CI}=1-k/k_{\mathrm{ctr}},$$ (1)

where k is the tumor regrowth factor for each group and $k_{\mathrm{ctr}}$ is the regrowth factor for the control group, which received no injection of BPD and light illumination.

### 2.8 Reactive Oxygen Species Explicit Dosimetry

PDT process can be described by a set of kinetic equations that can be simplified to describe the creation of ${\left[\mathrm{ROS}\right]}_{\mathrm{rx}}$.^8^^,^^24^^,^^25^ These equations are dependent on the temporal and spatial distribution of ϕ, photosensitizer concentration ($\left[S_{0}\right]$), ground state oxygen concentration ([O23]), oxygen supply rate (g), and the photosensitizer-specific reaction-rate parameters (δ, β, σ, and ξ). The relevant equations are

d[S0]dt=−[O23][O23]+β([S0]+δ)ϕ[S0]ξσ, (2)

d[O23]dt=−[O23][O23]+βϕ[S0]ξ+g(1−[O23][O23]0), (3)

d[ROS]rxdt=ξ[O23][O23]+βϕ[S0]. (4)

The details of the five parameters involved in the kinetic equations can be found elsewhere (see Table 1).^18^^,^^19^ ξ is the photochemical oxygen consumption rate per light fluence rate and photosensitizer concentration under ample O23 supply. σ is the probability ratio of an ROS molecule to react with a ground state photosensitizer compared to the ROS molecule reacting with a cellular target. β represents the ratio of the monomolecular decay rate of the triplet state photosensitizer to the bimolecular rate of the triplet photosensitizer quenching by O23. δ is the low-concentration correction factor, and g is the maximum macroscopic oxygen perfusion rate. ${\left[\mathrm{ROS}\right]}_{\mathrm{rx}}$ was calculated by integrating the term of the right-hand side of Eq. (4) over the time course of PDT treatment using the measured ϕ, $\left[S_{0}\right]$, and [O23]:

[ROS]rx=∫0Tξ[O23][O23]+βϕ[S0]dt. (5)

${\left[\mathrm{ROS}\right]}_{\mathrm{rx}}$ is determined at 3-mm depth using the calculated ϕ and measured [O23] concentration at 3-mm depth to ensure that its minimum value covers the maximum extent of RIF tumors used in this study. $\left[S_{0}\right]$ is assumed to be uniform throughout the tumor. If one uses 1 or 2 mm instead of 3 mm, the value of ${\left[\mathrm{ROS}\right]}_{\mathrm{rx}}$ will increase, thus the resulting threshold ${\left[\mathrm{ROS}\right]}_{\mathrm{rx}}$ value but the general curve shape [Fig. 6(c)] will not change.

## 3 Results

BPD-mediated PDT with different in-air fluences and different $\phi_{\text{air}}$, and different exposure times was performed in mouse models bearing RIF tumors. Light fluence rate, photosensitizer concentration, and tissue oxygenation were measured to calculate PDT dose and ${\left[\mathrm{ROS}\right]}_{\mathrm{rx}}$. Table 2 summarizes all treatment conditions as well as the measured and calculated quantities using the photochemical parameters summarized in Table 1.

**Fig. 3:**
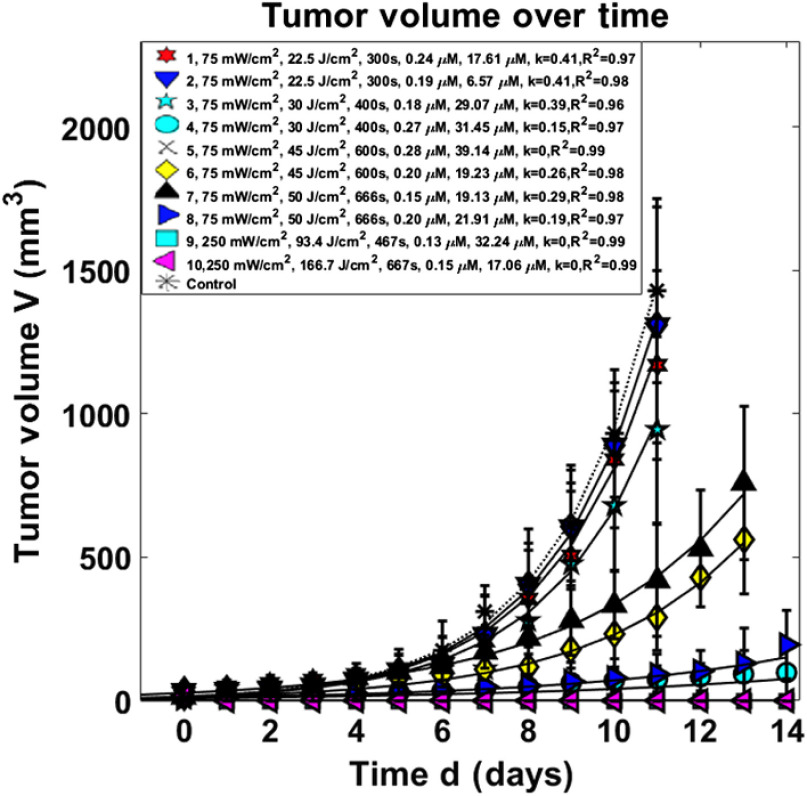
Tumor volumes over days after $V=V_{0}e^{kd}$ PDT treatment. Solid lines are the exponential fit to the data with a functional form of $e^{kd}$, where d is days ater PDT treatment. The resulting tumor regrowth rates k and its uncentrainty δk are listed in Table 2. The legend for each group lists: in-air fluence rate, in-air fluence, treatment time (in s), BPD concentration (in *μ*M), tissue oxygen concentration (in *μ*M), tumor regrowth rate, and $R^{2}$ of the fitting the the exponential equation.

To compare the regrowth rate between different tumors, volumes were normalized so that the initial volumes on day 0 were matched to be the same among all tumors, $∼12\;{\mathrm{mm}}^{3}$. Figure 3 shows the normalized tumor volume versus time (in days) for the 10 treatment groups and the control group along with the fits to the data with an exponential growth equation. These exponential fits to the data determine the value of k for each treatment group of mice. The statistical analyses showed a reduction of tumor regrowth rate for all treated groups compared to the control (all with p<0.05). Some mice within the same group had different CIs. For the group #4 with a total fluence of $30\;J/{\mathrm{cm}}^{2}$ and $\phi =75\;\mathrm{mW}/{\mathrm{cm}}^{2}$, one out of three (33.3%) mice showed a complete response (no tumor regrowth at 14 days post-PDT). This is reflected in Table 2 under the column labeled CI variation count.

**Fig. 4:**
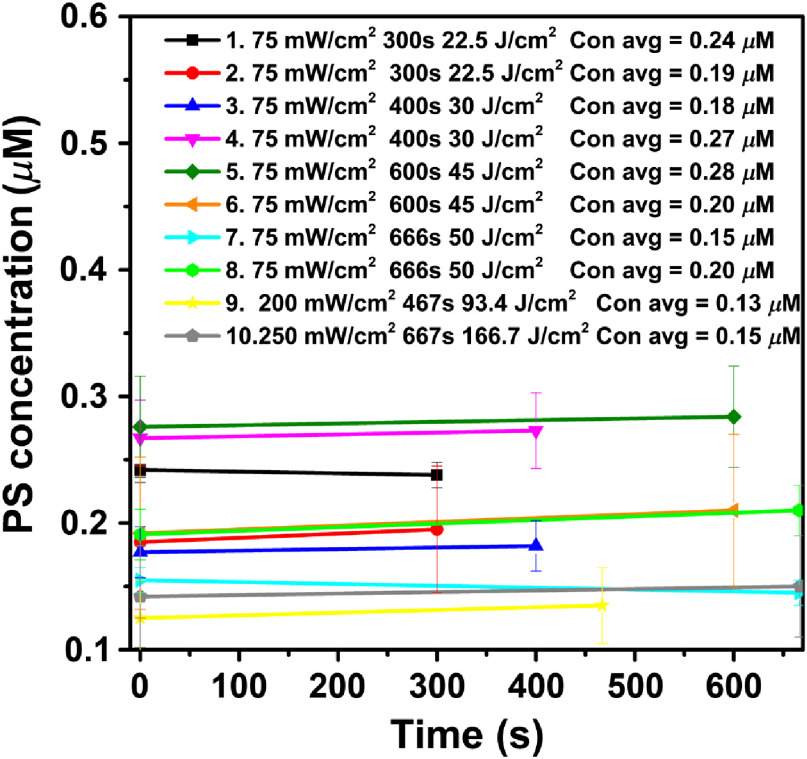
The temporal changes of BPD concentration versus time at 3-mm depth for the treatment conditions. The lines represent linear fits to BPD concentrations during treatment. The average PS concentration [PS] is given in the figure legend for each condition. The uncertainty of [PS] is listed in Table 2.

Figure 4 shows the temporal dependence of photosensitizer uptake.

**Fig. 5:**
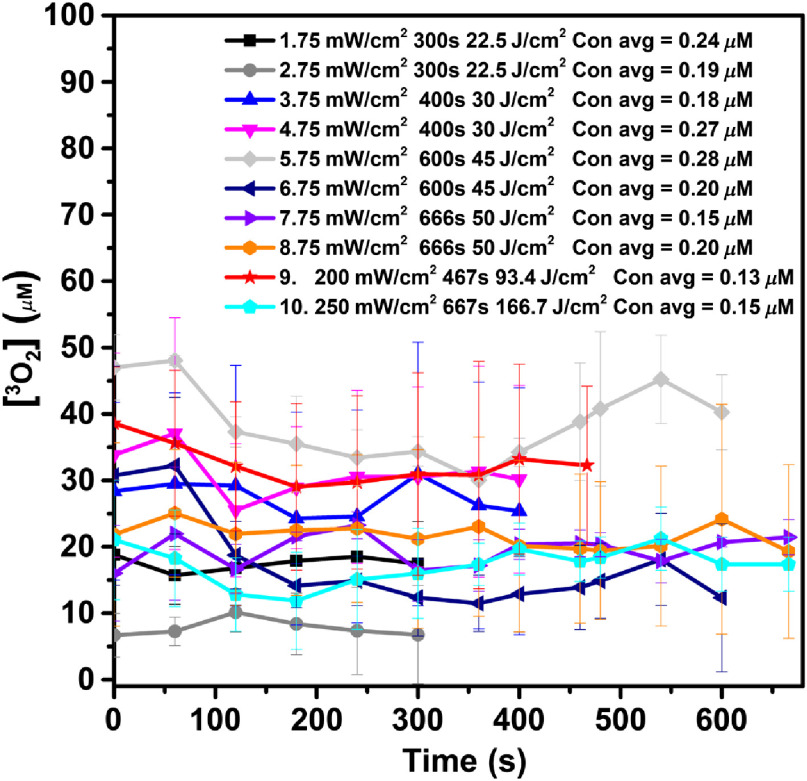
The temporal dependence of [O23] concentration for different treatment conditions. The concentration of [O23] and its uncertainty are listed in Table 2.

Measured [O23] was used to refine the photochemical parameters previously determined for the reactive oxygen species explicit dosimetry model used to calculate ${\left[\mathrm{ROS}\right]}_{\mathrm{rx}}$. Measured data of [O23] are shown with symbols in Fig. 5. There is no significant change in [O23] during the treatment.

**Fig. 6:**
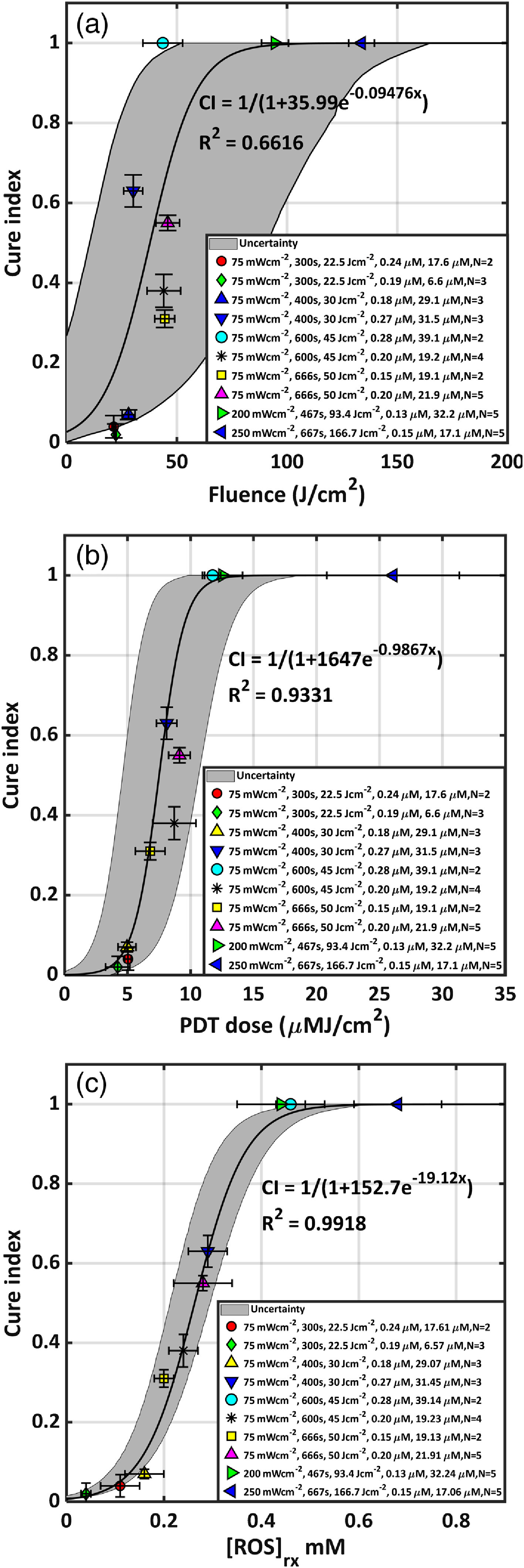
CI plotted against (a) fluence at a 3-mm tumor depth, (b) calculated PDT dose at 3-mm depth, and (c) mean reacted oxygen species at 3-mm depth (${\left[\mathrm{ROS}\right]}_{\mathrm{rx}}$) calculated using Eqs. (3)–(5) and the parameters summarized in Table 1. The solid lines show the best-fit to the data with functional forms $\mathrm{CI}=1/(1+35.99e^{-0.09476x})$, $1/(1+1647e^{-0.9867x})$, and $1/(1+152.7e^{-19.12x})$ with $R^{2}=0.6616$, 0.9331, and 0.9918 for (a), (b), and (c), respectively. The gray region indicates the upper and lower bounds of the fit with 95% confidence level. The gray curves are obtained from an MC simulation of the sigmoid model (see text for details).

Fluence, PDT dose, and calculated ${\left[\mathrm{ROS}\right]}_{\mathrm{rx}}$ at 3 mm were compared as dosimetric quantities to correlate with the treatment outcome of BPD-mediated PDT for RIF tumors on a mouse model. The outcome was evaluated by the calculation of CI. No tumor regrowth up to 14 days after treatment resulted in a CI of 1. PDT dose is calculated using the product of PS uptake and measured light fluence rate at 3 mm. We used Eq. (4) and the photophysiological parameters shown in Table 2 to calculate ${\left[\mathrm{ROS}\right]}_{\mathrm{rx}}$. The goodness of the fit and the corresponding upper and lower bounds of the fit with 95% confidence interval (gray area) to the fluence, PDT dose, and the calculated ${\left[\mathrm{ROS}\right]}_{\mathrm{rx}}$ are presented in Fig. 6. Figure 6(a) shows that, while fluence correlates sigmoidal with the PDT outcome, it exhibits large uncertainties as defined by the large bounds of the gray area, as well as by the low value of $R^{2}=0.6616$. As shown in Fig. 6(b), PDT dose allows for reduced subject variation and improved predictive efficacy as compared to fluence alone. PDT dose showed a better correlation with CI with a higher value of $R^{2}=0.9331$ and a narrower band of gray area as it accounts for both light dose and tissue [BPD] levels. However, PDT dose overestimates ${\left[\mathrm{ROS}\right]}_{\mathrm{rx}}$ in the presence of hypoxia as it does not account for the oxygen dependence of ROS quantum yield. The goodness of fit $R^{2}=0.9911$ and the narrowest gray area in Fig. 6(c) shows that the measured ${\left[\mathrm{ROS}\right]}_{\mathrm{rx}}$ correlates the best with CI. ${\left[\mathrm{ROS}\right]}_{\mathrm{rx}}$ accounts for the key quantities of light fluence, photosensitizer concentration, and tissue oxygen level, respectively.

## 4 Discussions

As shown in Fig. 2, the accuracy of the *in vivo* BPD concentration determined by our fluorescence spectroscopy is validated by comparing it with *ex vivo* measurements. *In vivo* fluorescence measurements were taken at 15 min and 3 h after BPD administration on the same tumors, and *ex vivo* fluorescence measurements were performed only once, at 3-h incubation time. The agreement between *in vivo* and *ex vivo* BPD concentration at 3 h is within 2%. Comparing the 15-min DLI *in vivo* BPD concentration with the 3-h DLI *ex vivo* BPD concentration, the *in vivo* measurements were about 11% lower, indicating a lower tissue uptake at shorter DLI.

Compared to control mice, all treated mice with total fluences larger than $30\;J/{\mathrm{cm}}^{2}$ had significant control of the tumor regrowth after PDT (see Fig. 3). The regrowth rate decreased when in-air light fluence increased (e.g., comparing the group of mice treated to $166.7\;J/{\mathrm{cm}}^{2}$ and that of $30\;J/{\mathrm{cm}}^{2}$). However, in-air light fluence alone is not a good predictor of the tumor regrowth because of significant variations of either BPD *in vivo* concentration or oxygen concentration ([O23]). As a result, we have split each (in-air fluence rate and in-air fluence) group further into subgroups depending on either BPD concentration or [O23], whenever there are a significant difference.

Based on a previous study,^26^ an empirical six-parameter fitting equation was used to fit the Monte-Carlo (MC) simulated data for a 1-cm diameter field, with $\mu_{a}=0.69\;{\mathrm{cm}}^{-1}$, and $\mu_{s}^{\prime }=11\;{\mathrm{cm}}^{-1}$.^19^ The equation is of the following form:^19^

$$\phi /\phi_{\text{air}}=\mathrm{INV}\cdot (1-b\cdot e^{-\lambda_{1}d})(C_{2}e^{-\lambda_{2}d}+C_{3}e^{-\lambda_{3}d}),$$ (6)

where the parameters $\lambda_{1}$ ($16.23\;{\mathrm{cm}}^{-1}$), $\lambda_{2}$ ($5.58\;{\mathrm{cm}}^{-1}$), $\lambda_{3}$ ($9.72\;{\mathrm{cm}}^{-1}$), b (0.37), $C_{2}$ (5.08), and $C_{3}$ (−0.03) are functions of $\mu_{a}(0.69\;{\mathrm{cm}}^{-1})$ and $\mu_{s}^{\prime }$ ($11\;{\mathrm{cm}}^{-1}$) and details of each can be found elsewhere.^26^ $\mathrm{INV}={\left[\mathrm{SSD}/(\mathrm{SSD}+d)\right]}^{2}$, where the source-to-surface distance (SSD)=9.34  cm based on the measurement of light fluence rate in water for the same collimated beam as a function of depth.

Figure 4 shows no variation of PS concentration during PDT for vascular BPD-mediated PDT. This is understandable because most of the BPD drug is located in the vasculature and PDT is obviously insufficient to deplete the BPD concentration $\left[S_{0}\right]$. Similarly, Fig. 5 shows that insignificant variation of [O23] concentration is observed for each group. Comparing the measured $\left[S_{0}\right]$ and [O23] with Eqs. (2) and (3), respectively, we come to the conclusion that d[S0]/dt=d[O23]/dt=0 for the vascular PDT condition. This is completely understandable because of the ample oxygen supply in the blood vessel and presumably ample BPD concentration in the vasculature.

Fluence, PDT dose at 3 mm, and ${\left[\mathrm{ROS}\right]}_{\mathrm{rx}}$ at 3 mm were compared as dosimetric quantities to estimate the outcome of BPD-mediated vascular PDT for RIF tumors on a mouse model. Outcome was evaluated by the calculation of CI. No tumor regrowth up to 14 days after treatment resulted in a CI of 1. The goodness of the fit and the corresponding upper and lower bounds of the fits (gray area) to the fluence, PDT dose, and mean ${\left[\mathrm{ROS}\right]}_{\mathrm{rx}}$ are presented in Fig. 6. MC simulation was used to produce the gray area due to the uncertainty in the parameter a, b for the sigmoid curve y=1/[1+a×exp(−bx)]. The means and standard deviations of the simulation parameters a and b were obtained using the global optimization toolbox of MATLAB^®^ (cftool.m). In the MC simulation, we selected 1000 parameter pairs (a,b) within the standard deviation (δa,δb) using a random number generator with normal distributions, the resulting calculated y=1/[1+a×exp(−bx)] for all (a,b) for a particular dosimetric metric (fluence, PDT dose, or ${\left[\mathrm{ROS}\right]}_{\mathrm{rx}}$) was used to generate a cumulative probability distribution for each of the y values between [0, 1]. We then found corresponding x values for the 2.5% tiles and 97.5% tiles of the cumulative probability distribution and they form the two bounds of the gray zone, the left and right bounds were joined to form the uncertainty (gray) areas. Figure 6(a) shows that, while fluence correlates following a sigmoid curve with PDT outcome, it exhibits large uncertainties as defined by the large bounds of the gray area as well as by the low value of $R^{2}=0.66$. As shown in Fig. 6(b), PDT dose allows for reduced subject variation and improved predictive efficacy as compared to fluence. PDT dose showed a better correlation with CI with a higher value of $R^{2}=0.93$ and a narrower band of gray area as it accounts for both light dose and tissue [BPD] levels. However, PDT dose overestimates ${\left[\mathrm{ROS}\right]}_{\mathrm{rx}}$ in the presence of hypoxia as it does not account for the oxygen dependence of ROS (mostly O12) quantum yield. The goodness of fit $R^{2}=0.99$ and the narrowest gray area in Fig. 6(c) shows that the mean ${\left[\mathrm{ROS}\right]}_{\mathrm{rx}}$ correlates the best with CI among the three.

Based on the findings of this study, PDT dose and ${\left[\mathrm{ROS}\right]}_{\mathrm{rx}}$ exhibit threshold dose behavior as they can be fitted by a sigmoid function $\left\{S(x)=1/(1+e[-(x-x_{0})/w_{0}]\right\}$, where $x_{0}=7.5\;\mu M\;J/{\mathrm{cm}}^{2}$ with uncertainty $w_{0}=1.0\;\mu M\;J/{\mathrm{cm}}^{2}$ and $x_{0}=0.26\;\mathrm{mM}$ with uncertainty $w_{0}=0.05$ for PDT dose and ${\left[\mathrm{ROS}\right]}_{\mathrm{rx}}$, respectively. For PDT dose, $x_{0}$ can be converted to the absorbed dose by BPD by multiplying the extinction coefficient ($\varepsilon =0.0783\;\mu M^{-1}\;{\mathrm{cm}}^{-1}$), resulting in $0.59\;J/{\mathrm{cm}}^{3}$, which corresponds to $(2.0\pm 0.2)\times 10^{18}\text{ photons}/{\mathrm{cm}}^{3}$ (by dividing the energy per photon $hc/\lambda =2.88\times 10^{-19}\;J$ for λ=690  nm). The mean PDT dose threshold for BPD at DLI 15 min ($7.5\;\mu M\;J/{\mathrm{cm}}^{2}$) is 7.7 times lower than those reported for BPD at DLI 3 h ($58\;\mu M\;J/{\mathrm{cm}}^{2}$).^19^ The mean ${\left[\mathrm{ROS}\right]}_{\mathrm{rx}}$ threshold concentration of $x_{0}=0.26\pm 0.05\;\mathrm{mM}$ for DLI of 15 min is 3.8 times lower than to the published result for BPD (0.98 mM) for DLI of 3 h.^19^ The decrease for PDT dose can be explained by a decrease of BPD uptake between the tissue and the vasculature, i.e., our result implies that BPD concentration in the vessel is 7.7 times higher than those in the tissue. This is consistent with the published literature where a simulation using a diffusing model showed much lower concentration of BPD in the vessel than that in the surrounding tissue.^27^ The decrease for ${\left[\mathrm{ROS}\right]}_{\mathrm{rx}}$ threshold dose for vascular BPD PDT is due to the fact that both BPD concentration and [O23] in the vessel are substantially higher than those in the surrounding tissue. However, to estimate the resulting [O23] difference between the vessel and the surrounding tissue requires an estimate of the light fluence inside the vessel versus the light fluence rate inside tissue. Some literature reports the former to be lower than the latter by 20%.^28^

## 5 Conclusion

The response of mouse RIF tumors to PDT depends on the tissue oxygenation, photosensitizer uptake, total energy delivered, and the ϕ, in which the treatment is delivered. An accurate dosimetry quantity for the evaluation of the treatment outcome should account for all of these parameters. This study evaluated the efficacy and outcomes of different PDT treatments and how fluence, PDT dose, and ${\left[\mathrm{ROS}\right]}_{\mathrm{rx}}$ compare as dosimetric quantities. The correlation between CI and ${\left[\mathrm{ROS}\right]}_{\mathrm{rx}}$ suggests that ${\left[\mathrm{ROS}\right]}_{\mathrm{rx}}$ at 3 mm is the best quantity to predict the treatment outcome for a clinically relevant tumor regrowth endpoint. PDT dose is a better dosimetry quantity when compared to fluence but is worse than ${\left[\mathrm{ROS}\right]}_{\mathrm{rx}}$ as it does not account for the consumption of [O23] for different ϕ. For BPD in RIF tumors, our measurements show constant temporal dependence of *in vivo* oxygen concentration during PDT, which cannot be well modeled by our macroscopic model; thus it is necessary to make [O23] measurements during PDT to obtain ${\left[\mathrm{ROS}\right]}_{\mathrm{rx}}$. We find the threshold value of ${\left[\mathrm{ROS}\right]}_{\mathrm{rx}}$ for BPD-mediated vascular PDT at DLI 15 min to be 3.8 times smaller than the corresponding value for BPD-mediated PDT at DLI 3 h. This is being reported for the first time.
